# Energy metabolism regulated by HDAC inhibitor attenuates cardiac injury in hemorrhagic rat model

**DOI:** 10.1038/srep38219

**Published:** 2016-12-02

**Authors:** Qiyuan Kuai, Chunyan Wang, Yanbing Wang, Weijing Li, Gongqing Zhang, Zhixin Qiao, Min He, Xuanlin Wang, Yu Wang, Xingwei Jiang, Lihua Su, Yuezhong He, Suping Ren, Qun Yu

**Affiliations:** 1 Department of Blood Products and Substitutes, Beijing Institute of Transfusion Medicine, Beijing, China; 2 Poisoning and Treatment Department, Beijing 307 Hospital, Beijing, China; 3 Key Laboratory for Molecular Enzymology and Engineering, the Ministry of Education, Jilin University, Changchun, China

## Abstract

A disturbance of energy metabolism reduces cardiac function in acute severe hemorrhagic patients. Alternatively, adequate energy supply reduces heart failure and increases survival. However, the approach to regulating energy metabolism conductive to vital organs is limited, and the underlying molecular mechanism remains unknown. This study assesses the ability of histone deacetylase inhibitors (HDACIs) to preserve cardiac energy metabolism during lethal hemorrhagic injury. In the lethally hemorrhagic rat and hypoxic myocardial cells, energy metabolism and heart function were well maintained following HDACI treatment, as evident by continuous ATP production with normal cardiac contraction. Valproic acid (VPA) regulated the energy metabolism of hemorrhagic heart by reducing lactate synthesis and protecting the mitochondrial ultrastructure and respiration, which were attributable to the inhibition of lactate dehydrogenase A activity and the increased myeloid cell leukemia-1 (mcl-1) gene expression, ultimately facilitating ATP production and consumption. MCL-1, the key target of VPA, mediated this cardioprotective effect under acute severe hemorrhage conditions. Our results suggest that HDACIs promote cardioprotection by improving energy metabolism during hemorrhagic injury and could therefore be an effective strategy to counteract this process in the clinical setting.

Hemorrhage, shock and multi-organ dysfunction lead all other etiologies of early mortality in traumatized patients, with approximately 400,000 deaths worldwide each year^1^,^2^,^3^,^4^. They are commonly associated with cell damage and metabolic disturbance; however, the mechanisms involved in these processes remain unclear. Myocardial energy metabolism is required for normal cardiac contractile function, which is vital for humans to maintain normal cell metabolism and a relatively stable internal environment. Regulating energy metabolism in favor of the impaired heart reduces heart failure and sustains life for an extended time, therefore decreasing multi-organ failure-associated morbidity^5^.

The disturbance of cardiac function and energy metabolism has been reported to play a vital role in hypoxic- and hemorrhagic-induced injury^6^,^7^. Previous evidence suggests that supplemental energy production increases survival^8^,^9^,^10^. Adenosine triphosphate (ATP), mainly synthesized in the mitochondria by the tricarboxylic acid (TCA) cycle, is an essential and direct source of energy for normal cardiac contraction, thus it is a promising indicator of heart failure and death^11^. We therefore sought to evaluate energy metabolism as a predictor of survival during hemorrhagic heart injury.

Histone deacetylase inhibitors (HDACIs), such as valproic acid (VPA) and suberoylanilide hydroxamic acid (SAHA), have been reported to offer considerable protection during experimental hemorrhagic shock. Although the precise molecular mechanism of HDACIs is still under investigation, their protective effect is mediated at least in part by their epigenetic regulation of protein acetylation^3^,^12^,^13^. Protein lysine acetylation is a key posttranslational epigenetic modification. Extensive studies during the past four decades have identified important roles for lysine acetylation in cellular regulation, particularly in the regulation of energy metabolism. Indeed, many metabolic enzymes are known to be acetylated^14^, and nearly all enzymes involved in energy metabolism, such as glycolysis, gluconeogenesis, the TCA cycle, fatty acid oxidation, glycogen metabolism and oxidative phosphorylation, are acetylated.

In this study, we used the protective effects of VPA administrated after hemorrhage to investigate the role of energy metabolism regulation in hemorrhagic-induced cardiac injury. During the period of hemorrhagic decompensation, energy metabolism and heart function were well maintained, and overall mortality was improved. Most importantly, MCL-1, an anti-apoptosis protein residing in the mitochondria^15^, was found to mediate cardioprotective activity by promoting energy metabolism after hemorrhagic cardiac injury.

## Results

### VPA treatment improves survival in severe hypoxic H9c2 cells and hemorrhagic rats

Hypoxia and free radical injuries are the main causes of acute heart function failure raised by acute severe hemorrhage. We evaluated the cytoprotective effects of three clinically available HDACIs on H9c2 cells; in hypoxic and oxidative stress models (Supplementary Fig. S1). H9c2 was chosen because it was the only commercial cell line derived from rat heart tissue with no transfection and could be subcultured *in vitro*. Moreover, H9c2 was previously reported to be energetically similar to primary cardiomyocytes and suggested as an *in vitro* model to mimic the responses of primary cardiomyocytes to hypoxia and oxidative stress^16^. After comprehensive comparison in two damage models (hypoxia and free radical damages), VPA showed the best cell protection compared with the other two drugs, especially under H_2_O_2_-induced free radical injury. We therefore chose VPA for the following experiments.

**Figure 1:**
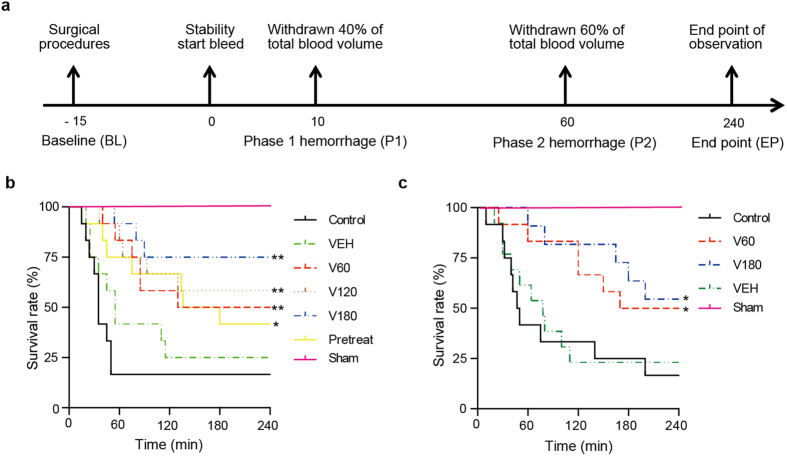
Effect of HDACIs treatment in two hemorrhagic animal models. (**a**) Timeline of the hemorrhagic surgery. Kaplan-Meier plot shows percentage of survival rate in the first hemorrhage model of 10 min (P1) treatment groups (**b**) and the second hemorrhage model of 1 h (P2) treatment groups (**c**). *P* < 0.05 versus control.

Two rat models for experimental hemorrhagic injury were employed to evaluate the therapeutic effects of VPA on lethal hemorrhage. In rat lethal hemorrhage models with 60% total blood loss (TBL), VPA was injected directly into the femoral vein following the onset of hemorrhage, allowing us to assess time-related differences in outcome. Experiments were performed according to the timeline described in Fig. 1a. For the type I model, VPA was administered 10 minutes after the start of hemorrhage (40% TBL, P1). Compared with the control group, the VEH group showed no significant differences, but the survival rates of VPA treatment increased significantly (Fig. 1b). VPA treatment with doses of 180 mg/kg, 120 mg/kg and 60 mg/kg resulted in survival rates of 75.0% (*P* = 0.0004), 58.0% (*P* = 0.0033) and 50.0% (*P* = 0.0057), respectively, while a 41.7% (*P* = 0.0407) survival rate was observed in the pretreatment group. VPA increased the 4-hour survival rate 4.5-fold (16.7% untreated survival versus 75.0% V180-treated survival). For the type II model, the time for administration of VPA was extended to 1 hour following initial hemorrhage (P2). In that model, the VEH group still showed no significant differences in comparison with the control group (Fig. 1c). However, VPA treatment showed a significant protective effect over the untreated control, increasing the 4-hour survival rate by 2.9-fold (17% untreated survival versus 50% V60-treated survival, *P* = 0.0367) and 3.4-fold (17% untreated survival versus 58% V180-treated survival, *P* = 0.0130). However, for each dose applied, the therapeutic effects were not as strong as those of groups with treatment at 10 minutes post-hemorrhage. To explore the protective mechanism of HDACIs to hypoxic injury in the early stage of hemorrhage, tissues of the type I rat model and the CoCl_2_-induced hypoxic model were chosen for further analysis.

Collectively, HDACI-treatment groups showed a significant improvement in 4-hour survival compared with control groups, and survival rates appeared to be dose-dependent.

### VPA regulated energy metabolism of hemorrhagic heart

**Figure 2:**
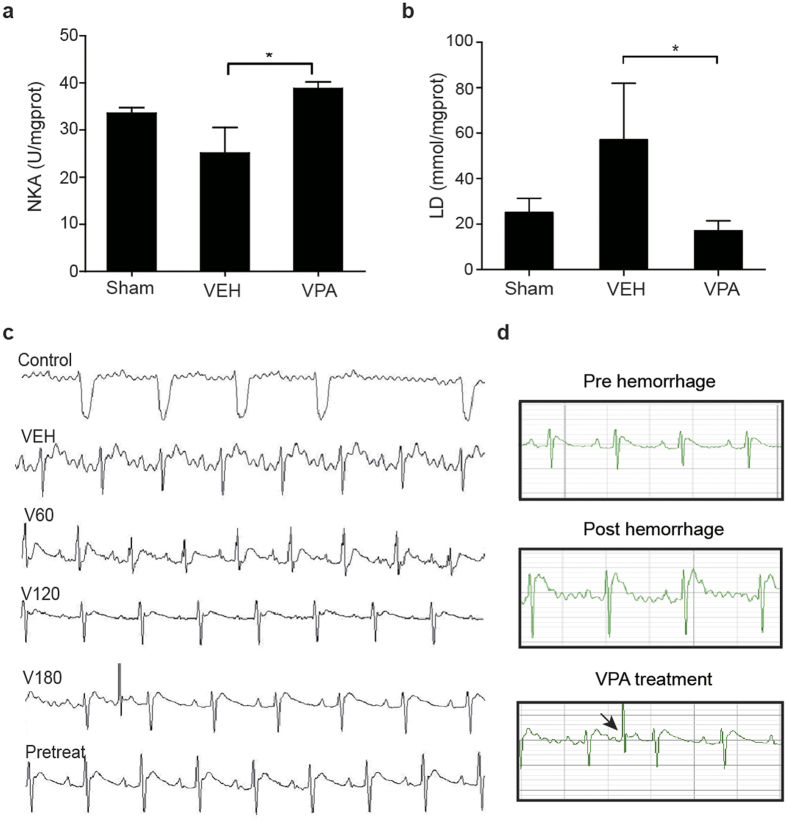
Effect of VPA treatment on energy metabolism of hemorrhagic heart. (**a**,**b)** Effect of VPA on NKA activity and lactate production in hemorrhagic rat models (n = 4). Values are reported as means ± SD. *P < 0.05 VPA versus VEH. (**c**) Representative ECG readings for each group after hemorrhage with and without VPA. (**d**) Amelioration of heart rhythm disorder with V180 treatment. The arrow shows the recovery of rhythm.

**Figure 3:**
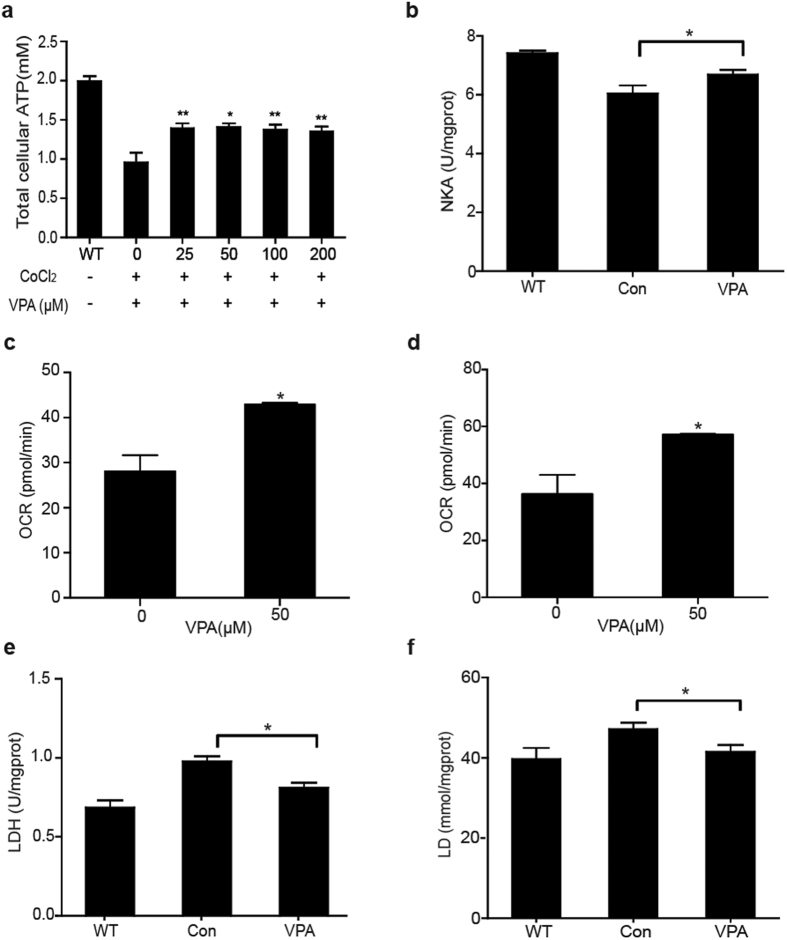
VPA regulated energy metabolism of hypoxic myocardial cell models. (**a**) Effect of VPA treatment on ATP production (n = 3). Values are reported as means ± SD. **P < 0.01 VPA versus 0 control. (**b**) Effect of VPA on NKA activity. H9c2 cells in hypoxia incubator for 24 hours with VPA (n = 5). Values are reported as means ± SD. *P < 0.05 VPA versus control. (**c,d**) VPA-mediated efficient oxygen consumption during hypoxic injury in H9c2 cells (n = 3). Values are reported as means ± SD. *P < 0.05 VPA versus 0 control. (**e,f**) Effect of VPA on LDH activity and lactate production in hypoxic H9c2 cells (n = 4). Values are reported as means ± SD. *P < 0.05 VPA versus control.

Histological features of rat heart tissue and other organ sections taken at the endpoint of observation were morphologically normal (Supplementary Fig. S2), indicating that the organs had not yet entered an irreversible phase of damage shortly after hemorrhage. After VPA treatment, energy metabolism was enhanced in hemorrhagic rat models, as evident by an increase of ATP consumption via an increase of Na-K-ATPase activity (Fig. 2a) and a decrease of lactic acid production (Fig. 2b), consistent with that of hypoxic myocardial cell models (Fig. 3b,f). Furthermore, ischemia-related arrhythmia was reduced upon VPA treatment while energy supply improved (Fig. 2c,d). For each of the first model groups, excluding the pretreatment group, an initial cardiac rhythm disorder (idiofocal tachycardia) appeared at 10 minutes. The onset of hemorrhagic arrhythmias occurred at a similar time point for each group and lasted until the end of observation in control and VEH groups. Arrhythmias observed in the HDACI treatment groups partially reversed over time and were significantly less pronounced than those in the control and VEH groups. Arrhythmias observed in the V60 group showed slight amelioration, but those in the V120 and V180 groups were reversed at 76.8 ± 7.6 minutes and 75.5 ± 9.4 minutes, respectively. The pretreatment group also displayed a pronounced anti-arrhythmic effect. VPA treatment increased MAP and decreased the H/S index compared with untreated controls (Supplementary Fig. S3).

A significant rise of total cellular ATP was observed in VPA-treated hypoxic H9c2 cells (Fig. 3a). Both ATP consumption (Na-K-ATPase activity, Fig. 3b) and mitochondrial respiration (Fig. 3c,d), including basal and maximal respiration, increased after VPA administration compared with control groups. Cell glycolysis was also changed upon HDACI treatment. In hypoxia cell cultures treated with VPA, LDH activity and lactate levels were significantly decreased (Fig. 3e,f). Energy analysis *in vitro* indicated that a more efficient energy production pathway other than glycolysis, most probably aerobic energy metablism, was induced in the hemorragic heart upon VPA treatment.

### VPA administration increases MCL-1 expression and ATP production

**Figure 4:**
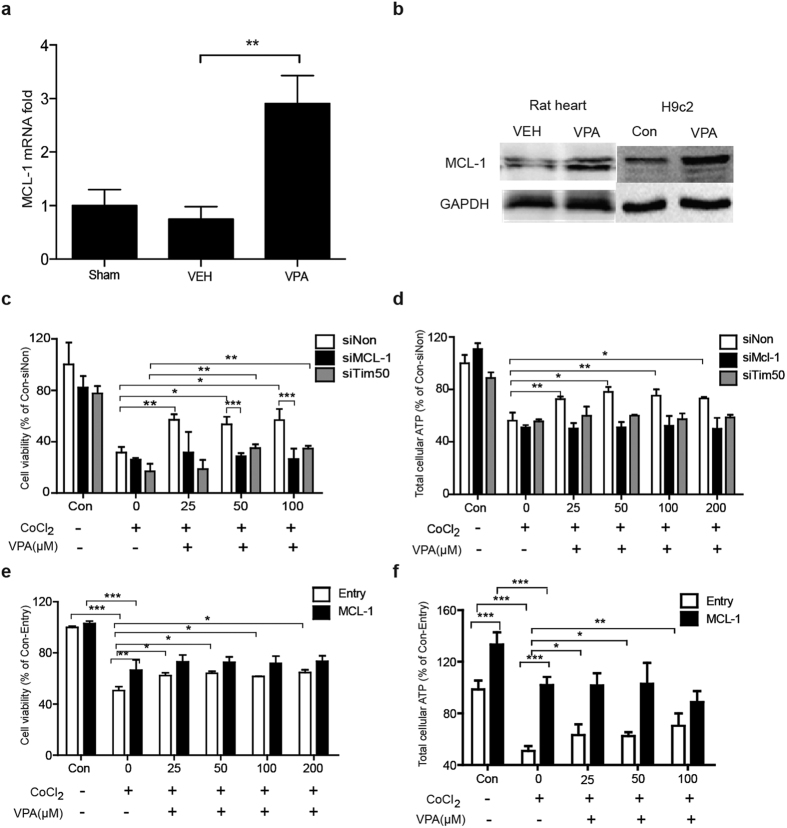
Effect of VPA treatment on MCL-1 expression and ATP production. (**a**) MCL-1 gene expression in Sham, VEH and VPA-treated rat heart tissues (n = 3). Values are reported as means ± SD. **P < 0.01 VPA versus control. (**b**) MCL-1 protein expression in rat heart tissue and hypoxic H9c2 cells. (**c**) VPA-mediated cytoprotection against hypoxic injury in non-targeting, MCL-1 and Tim50-gene silenced H9c2 cells (n = 3). Values are reported as means ± SD. *P < 0.05, **P < 0.01. (**d**) Cellular ATP production in non-targeting, MCL-1, Tim50-gene silenced H9c2 cells (n = 3). Values are reported as means ± SD. *P < 0.05, **P < 0.01. (**e**) VPA-mediated cytoprotection against hypoxic injury in pCMV6-AC-MCL-1 or Entry transfected H9c2 cells (n = 3). Values are reported as means ± SD. *P < 0.05, **P < 0.01, ***P < 0.001. (**f**) Cellular ATP production in pCMV6-AC-MCL-1 or Entry transfected H9c2 cells. Values are reported as means ± SD. *P < 0.05, **P < 0.01, ***P < 0.001.

We further explored the effects of HDACI on aerobic energy metablism and its related mechanism. An isoform of MCL-1 imported into the mitochondrial matrix by translocation of inner mitochondrial membrane 50 (Tim50) was recently reported to be necessary to facilitate ATP production and mitochondrial respiration^17^. We thus analyzed MCL-1 mRNA and protein and found them to be significantly increased after VPA treatment in the heart of hemorragic rats compared with the VEH group (Fig. 4a,b), while Tim50 mRNA and protein was not changed significantly after VPA treatment in the heart of hemorragic rats (data not show).

The MCL-1 protein in the hypoxic H9c2 cells was also elevated after VPA treatment, which was in accodence with the tissues (Fig. 4b).

To assess the ability of MCL-1 to mediate the protective activity of HDACI, RNA-mediated interference (RNAi) gene silencing of MCL-1 and Tim50 was used to prevent MCL-1 expression or import it into the mitochondrial matrix (Supplementary Fig. S4a,b). Cell viability assays revealed that non-targeting control siRNA-transfected H9c2 cells treated with VPA were protected from CoCl_2_-induced hypoxic injury (Fig. 4c). In contract, MCL-1 gene silencing arrested the VPA protective effect, while Tim50 gene silencing did not have the same effect on VPA-mediated survival. Collectively, these data indicated that HDACI improved cell survival against hypoxic injury through the activity of MCL-1 located at the outer mitochondrial membrane. Meanwhile, both MCL-1 and Tim50 gene silencing prevented ATP production from increasing after VPA treatment (Fig. 4d), indicating that VPA improved total cellular ATP production during hypoxic injury through the activity of MCL-1 located in the mitochondrial matrix. Plasmid-transfection of MCL-1 was used to increase MCL-1 expression using pCMV6-AC-MCL-1 vector to verify these results (Supplementary Fig. S4c). PCMV6-Entry-transfected cells were used as a negative control. The results showed that MCL-1 overexpression facilitated both cell viability and ATP production compared to the controls (Fig. 4e,f). The ability of MCL-1 to mediate the protective effect of VPA was also assessed *in vivo*. Compared with the vehicle control, the survival time in the group treated with MCL-1 inhibitor was reduced significantly (Supplementary Fig. S5).

### MCL-1 maintains the mitochondrial integrity and respiration of hypoxic myocardial cells

**Figure 5:**
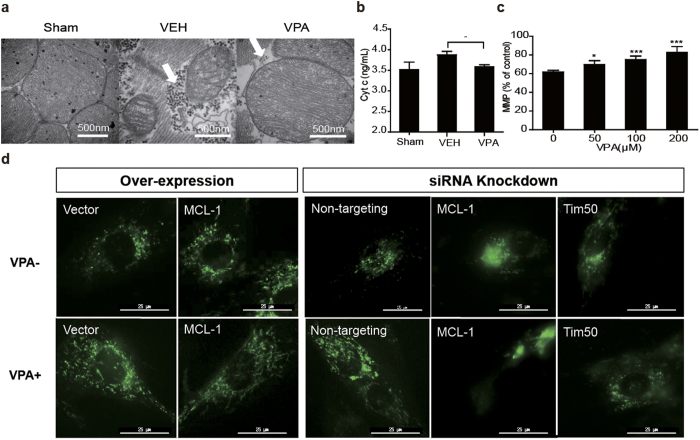
The effect of MCL-1 for maintaining the integrity of mitochondria. (**a**) Representative tracing of cardiac mitochondria. Heart tissues were obtained after 1 hour of 40% hemorrhage (VEH: 0.9% saline; VPA: 180 mg/kg), scale bar = 500 nm. (**b**) Release of cyt c from mitochondrial fractions. 180 mg/kg VPA was treated (n = 3). Values are reported as means ± SD. *P < 0.05 VPA versus VEH. (**c**) MMP measured in H9c2 cells (n = 3). Values are reported as means ± SD. *P < 0.05, ***P < 0.001 VPA versus 0 control. (**d**) The mitochondrial image of live H9c2 cells. Pictures were taken at 8 h after hypoxic injury. Left: Empty vector or pCMV6-AC-MCL-1 transfected H9c2 cells were treated with (VPA+) or without (VPA−) VPA. Right: Non-targeting, MCL-1 or Tim50-gene silenced H9c2 cells were treated with (VPA+) or without (VPA−) VPA.

Mitochondria are essential for cell growth, efficient oxidative phosphorylation and ATP production. We thus analyzed the ultrastructure of cardiac mitochondria, release of cyt *c* of rat hearts. As shown in Fig. 5a, abundant mitochondria were aligned in parallel in normal rat hearts, and double-lay membranes were readily discernible. Ultrastructure defects, including damaged mitochondrial membrane and cristae structure and glycogen accumulation in the cytoplasm, were apparent in most mitochondria in the VEH group. In the treatment group, the integrity of mitochondrial membranes and cristae structures was maintained, glycogen accumulation disappeared, and there were no morphological alterations such as loss of myofibrils or cytoplasmic vacuolization. Cyt *c* is an electron transfer body involved in oxidative phosphorylation, and its release from the mitochondrion signals mitochondrial membrane damage. Release of cyt *c* in the V180 group was significantly decreased compared with the controls (Fig. 5b). These data suggested that regulation of aerobic metabolism could prevent mitochondrial apoptosis under hypoxic conditions induced by severe hemorrhage.

Lastly, mitochondrial membrane potential (MMP) was detected in hypoxic H9c2 cells. The result showed that MMP was increased in VPA-treated hypoxic H9c2 cells compared with controls, confirming the protective effective of HDACI on mitochondrial membrane integrity (Fig. 5c).

To confirm the critical effect of Mcl-1 for maintaining mitochondrial integrity, live H9c2 cell mitochondrial activity was dynamically observed using a DeltaVision Microscopy Imaging System. VPA significantly protected H9c2 cell mitochondria against hypoxic injury compared with controls (Fig. 5d left, Supplementary Video S1, 2). Over-expression of MCL-1 gene displayed a similar protective effect even without VPA treatment (Supplementary Videos S3 and 4). In contrast, MCL-1 gene silencing arrested the VPA-mediated protective effect (Fig. 5d right, Supplementary Video S5,S6,S7,8). Blockage of mitochondrial localization of MCL-1 isoform by Tim50 silencing resulted in similar consequences (Supplementary Video S9, 10). These findings confirmed that MCL-1 played an important role in maintaining mitochondrial integrity and activity in hypoxic myocardial cells.

**Figure 6:**
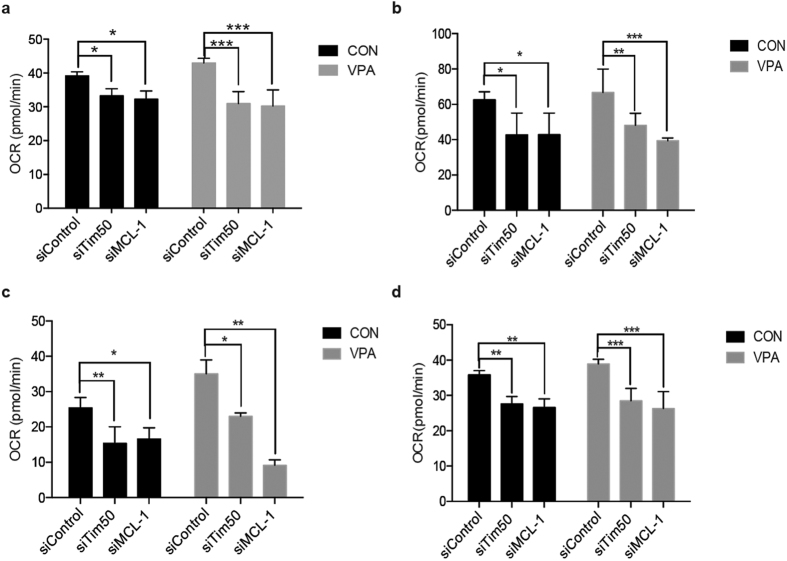
Mitochondrial respiration of hypoxic H9c2 cells. Oxygen consumption rate (OCR) was measured with or without MCL-1 and Tim50 gene knockdown respectively in the H9c2 cells. Basal respiration (**a**), maximal respiration (**b**), Spare respiration (**c**), and ATP production (**d**) are shown (n = 3). Values are reported as means ± SD. *P < 0.05, **P < 0.01, ***P < 0.001.

We also determined whether mitochondrial respiration was altered by measuring oxygen consumption rates (OCRs) using the Seahorse XF^e^96 analyzer. MCL-1 or Tim50 deletion significantly decreased basal respiration, maximal respiration and spare respiration capacity of hypoxic H9c2 cells when compared with siControls (Fig. 6a–c); mitochondrial respiration could not be restored with VPA treatment. Lastly, compared with non-targeting siRNA-transfected H9c2 cells, ATP production in MCL-1 and Tim50 knockdown cells was significantly decreased in control and VPA treatment groups (Fig. 6d). Collectively, these data indicate that MCL-1 is required for optimal oxidative phosphorylation.

## Discussion

Pathophysiological changes following severe hemorrhage include reduced blood circulation, tissue hypoperfusion, microcirculation disorder and cell hypoxia. Pharmacologic interventions can protect cardiac cells from hypoxia at the very early stages of hemorrhage and sustain the viability and function of the heart. We hypothesized that the regulation of cardiac energy metabolism would alleviate cardiac dysfunction and prolong survival when oxygen availability is low. Hypoxic and hemorrhagic injury is linked to the acquisition of aberrations in protein acetylation through histone acetyltransferases (HATs) and HDACs^18^. Nearly all metabolic enzymes involved in glycolysis and the TCA cycle are epigenetically modified by acetylation, especially enzymes involved in aerobic metabolism^14^,^19^. Recent data revealed that HDACI directly inhibits HDACs and may increase the survival of hemorrhagic patients by restoring the balance of acetylation^20^. Here, we found that regulation of energy metabolism by HDACIs can attenuate hypoxic and hemorrhagic cardiac injury.

Although systemic ATP production is reduced as a consequence of hypovolemia, the time frame over which the organs become irreversibly damaged is yet unclear. This information will be greatly helpful to optimize procedures for intervention and to prolong the period of time to irreversible shock following severe hemorrhage, potentially with the identification of a ‘checkpoint’ in the process of severe hemorrhagic injury; pharmacologic intervention prior to the checkpoint might improve survival. Notably, during the compensation period before the checkpoint, the systemic blood supply is redistributed to the organs most necessary for sustaining life, such as the heart and brain^21^. However, during the decompensation period following the checkpoint, the disease progresses to microcirculation disorder, multi-organ dysfunction and eventually death. Based on our data and that of others^3^,^22^,^23^, pharmacologic intervention within the first 10 minutes of initiation of hemorrhagic injury is optimal for preserving life^24^,^25^. Although heart failure is the final consequence of several different etiologies, impaired myocardial energy metabolism has been proposed as one common underlying process during severe hemorrhage^26^. ATP is required for both cell viability and myocardial pump function, specifically contraction and relaxation^11^. The positive effect of HDACI on energy metabolism of the heart is evident in our study, and the underlying mechanisms are construed in the following observations.

First, aerobic metabolism was enhanced by maintaining the integrity of the mitochondria. The integrity of some subcellular structures, particularly the mitochondrial membrane and interior cristae, of cardiomyocytes in animals with massive blood loss were well preserved, as verified by the changes of MMP^27^, cyt *c*^28^,^29^ and Na-K-ATPase^30^. In the myocardium, oxidative phosphorylation in the mitochondria is the primary means of ATP synthesis. Therefore, it would be evolutionally efficient to use TCA route for the energy supply in the case of emergent blood loss. Thus, we tried to dig into the molecular mechanisms relevant to aerobic metabolism with various models in attempt to better understand critical molecular events during the process.

Second, our data showed that HDACI treatment significantly increased MCL-1 expression. We observed MCl-1 was regulated by HDACI in normal tissue cells during hypoxia, suggesting that this gene may mediate the therapeutic effect of HDACI drugs during hypoxic injury. Furthermore, MCL-1 gene silencing disabled both the cytoprotective activity of VPA treatment and its ability to facilitate mitochondrial ATP production; however, MCL-1 overexpression facilitated both cytoprotective activity and ATP production, suggesting a key role of MCL-1 in the pharmacological effect of VPA and the pathogenesis of hemorrhage-induced organ dysfunction. Moreover, MCL-1 maintains the mitochondrial integrity and respiration of hypoxic myocardial cells confirmed by dynamic microscopy imaging and Seahorse mitochondrial stress analysis. Previous studies showed that MCL-1 on the outer mitochondrial membrane maintains mitochondrial integrity, and MCL-1 localized to the mitochondrial matrix promotes ATP production. Therefore, as shown in our study, MCL-1 facilitates mitochondrial homeostasis and supports mitochondrial bioenergetic function, which is essential for cell survival^17^.

Third, cellular anaerobic glycolysis was suppressed by regulating LDH. Specifically, LDH activity was reduced and lactate production was increased. LDH is a key metabolic enzyme that catalyzes the irreversible conversion of pyruvate into lactate and plays a central role in anaerobic glycolysis^31^. A lack of oxygen availability causes a cellular shift from aerobic to anaerobic metabolism with an increase in LDH and lactate that leads to lactic acidosis. LDH activity was decreased in the VPA treatment groups, being accompanied by the decreased production of lactate, which may partially explain the observed elevation of ATP production and drop of glycogen accumulation in the cytoplasm. In conclusion, the increased myocardial energy metabolism induced by VPA in our models of severe hemorrhagic injury was strongly correlated with improved animal survival.

To enhance the survival rate in the rat lethal hemorrhage models, our study has optimized the timing for the interventional approach of VPA treatment, providing a solid foundation for the translation of such a therapeutic strategy into human trials. It appears that the administration of the drug needs to be shifted to the time point closest to the onset of hypoperfusion during hemorrhagic injury. Pilot studies in rats showed that the survival rate depends on the timing of the administration of VPA. VPA treatment carried out 1 hour instead of 10 minutes after initial blood loss resulted in a significant drop in survival rate from 75% to 58% in the dosing group of 180 mg/kg body weight.

## Conclusions

Though VPA is an approved drug for epileptic seizures and is also used as a mood stabilizer in various neuropsychiatric conditions, its biological impact on the nervous system had not previously been examined. ‘In this study, we observed an interesting and meaningful therapeutic effect of VPA in its ability to reduce ischemia-related arrhythmia after acute severe hemorrhagic injury. The antiarrhythmic effect of VPA was found to remain in rats after the vagus nerve was cut and ganglion blocked, so this effect of VPA was thought to be unrelated to the central nervous system. Our study has clearly demonstrated the therapeutic benefits and the cardioprotective effect of HDACIs in hypoxic and hemorrhagic injury as well as revealed the underlying molecular pharmacological mechanisms of energy metabolism involving regulation of MCL-1 expression and the maintenance of mitochondrial integrity, respiration and ATP production. HDACIs are thus promising therapeutic candidates for acute hypoxic and hemorrhagic injury.

## Methods

Additional details are provided in Supplementary Methods.

### Cell damage and HDACI rescue

In the hypoxia model, rat cardiomyoblasts H9c2 were cultured with 2 mmol/L CoCl_2_ (Sigma-Aldrich, St. Louis, MO, USA) for oxygen deprival for 5 hours and then treated with one of the following HDACI clinical drugs, VPA (Sigma-Aldrich), SAHA (Selleckchem, Houston, TX, USA), chidamide (Chipscreen, Shenzhen, Guangdong, China) for 5 hours. To induce oxidative stress injury, cells were treated with 0.03% H_2_O_2_ for 15 minutes and then cultured with HDACI drugs for 24 hours.

Cell viability and ATP production were measured by MTT assay and CellTiter-Glo^®^ luminescent cell viability assay (Promega, Madison, WI, USA) respectively. Energy metabolism indictors, such as Na-K-ATPase activity, LDH activity, lactate production, mitochondrial respiration and mitochondrial membrane potential, were also assessed in rat H9c2 cells.

### Animal studies

All animal experiments were reviewed and approved by the Institutional Animal Care and Use Committee of Academy of Military Medical Science, Beijing, China (IACUC-2009-001), and the animals were housed and handled in accordance with the guidelines of the National Institutes of Health. Male Wistar rats were anesthetized and were continuously monitored with MP150 physiological recorder (Biopac, Goleta, CA, USA). Lethal hemorrhage was performed in two phases: 40% artery blood was rapidly withdrawn over 10 minutes (phase 1, P1), and another 20% vein blood loss (P2) over the next 50 minutes was to simulate the process of a chronic bleeding stage. The animals were allowed to recover after the hemorrhage stage.

Two kinds of hemorrhage models based on time point of VPA i.v. administration were used. The Type I model (n = 12 in each group), treated at the end of P1, was classified as: Sham, control, VEH, V60 (VPA 60 mg/kg), V120 (VPA 120 mg/kg), V180 (VPA 180 mg/kg) and pretreat (VPA 180 mg/kg, i.m.) groups. The Type II model (n = 11∼13), treated at the end of P2, was classified as: Sham, control, VEH, V60 and V180.

Several rat tissues were paraffin-embedded and stained for tissue histology. Na-K-ATPase activity, lactate production and cyt *c* content were assayed in heart tissues. The mitochondrial ultrastructure of heart tissues was imaged using a JEM-1200EX transmission electron microscope (JEOL, Tokyo, Japan)^32^.

### Loss-of-function and gain-of -function experiments

Gene knockdown of H9c2 cells was achieved by transfection with 60 nM siRNA specific for rat MCL-1 or Tim50 using Lipofectamine^®^ RNAiMAX Reagent (Invitrogen, Carlsbad, CA, USA). The MCL-1 over-expression-stable H9c2 cells were established by pCMV6-AC-MCL-1 plasmid (OriGene, Rockville, MD, USA) transfection and G418 screening.

The gene knockdown or over-expression of cells was verified by real-time RT-PCR and Western blot and then subjected to hypoxic injury for the following loss-of-function and gain-of-function experiments: cell viability, ATP production, DeltaVision microscopy imaging (GE, Pittsburgh, PA, USA) and oxygen consumption rates (OCRs, Seahorse, North Billerica, MA, USA).

### Statistics

All data are expressed as mean ± standard deviation (SD). Intergroup differences were analyzed by analysis of variance followed by Dunnett’s or Sidak’s multiple comparisons test. Statistical significance between two groups was calculated using the unpaired t test with Welch’s correction and animal survival time was analyzed by a log-rank test. A *P* value less than 0.05 was considered to be statistically significant.

## Additional Information

**How to cite this article**: Kuai, Q. *et al*. Energy metabolism regulated by HDAC inhibitor attenuates cardiac injury in hemorrhagic rat model. *Sci. Rep.* **6**, 38219; doi: 10.1038/srep38219 (2016).

**Publisher's note:** Springer Nature remains neutral with regard to jurisdictional claims in published maps and institutional affiliations.

## Supplementary Material

Supplementary Information

Supplementary Video 1

Supplementary Video 2

Supplementary Video 3

Supplementary Video 4

Supplementary Video 5

Supplementary Video 6

Supplementary Video 7

Supplementary Video 8

Supplementary Video 9

Supplementary Video 10
