# Supplementary material for: Energy metabolism regulated by HDAC inhibitor attenuates cardiac injury in hemorrhagic rat model
Source: Sci Rep. 2016 Dec 2;6:38219. doi: 10.1038/srep38219 (PMC5133557; doi:10.1038/srep38219)
Supplement: Supplementary Information [file srep38219-s1.pdf]

Energy metabolism regulated by HDAC inhibitor attenuates cardiac injury in hemorrhagic rat model

Qiyuan Kuai[1†], Chunyan Wang[1, 2†], Yanbing Wang[1, 3†], Weijing Li[1], Gongqing Zhang[1], Zhixin Qiao[1], Min He[1], Xuanlin Wang[1], Yu Wang[1], Xingwei Jiang[1], Lihua Su[1], Yuezhong He[1], Suping Ren[1*] and Qun Yu[1*]

[1] Department of Blood Products and Substitutes, Beijing Institute of Transfusion Medicine, Beijing, China

[2] Poisoning and Treatment Department, Beijing 307 Hospital, Beijing, China

[3] Key Laboratory for Molecular Enzymology and Engineering, the Ministry of Education, Jilin University, Changchun, China

Supplemetary Infomation

Supplemetary file 1: Figure S1. Effect of HDACIs treatment in cell hypoxia and oxidative stress models. (**a-c**) The myocardial protection of VPA, SAHA, chidamide was explored in the $CoCl_2$-induced hypoxia model. (**d-f**) The myocardial protection of VPA, SAHA, chidamide was explored in the H_2O_2-induced free radical model (n=3). Values are reported as means ± SD. **P* < 0.05, ***P* < 0.01 versus 0 control.

Supplemetary file 2: Figure S2. Histology of tissues at the endpoint of observation time in hemorrhagic rats. Representative histology views (H&E Stain) in VEH control and VPA-treated animals are shown, 100×.

Supplemetary file 3: Figure S3. H/S index and MAP in rats. (**a**) 60% of total blood was removed and the rat was treated at 60 minutes; the V180 rat (n=1) was treated with 180 mg/kg VPA at 10 minutes; the VEH rat (n=1) was treated with an equal volume of NS (normal saline, 0.9% NaCl); (**b**) 60% of total blood was removed in 60 minutes; the VPA group (n=4) was treated with 180 mg/kg VPA after 10 minutes from baseline (T=0); the VEH group (n=4) was treated with an equal volume of NS.

Supplemetary file 4: Figure S4. Knockdown and overexpression. (**a**, **b**) Gene and protein expression after RNA-mediated interference (RNAi)-gene silencing of MCL-1

and Tim50. Top: Relative gene expression was measured by qRT-PCR (n=3). Values are reported as means ± SD. *P < 0.05, **P < 0.01, ***P < 0.001 versus negative. Bottom: Protein expression was detected by Western blot. (**c**) pCMV6-AC-MCL-1 was overexpressed in H9c2 cells. Top: Relative gene expression was measured by qRT-PCR (n=3). Values are reported as means ± SD. **P < 0.01 versus Entry. Bottom: Protein expression was detected by western blot.

Supplemetary file 5: Figure S5. The ability of MCL-1 to mediate the protective activity of VPA. For the type I model, rats were treated with vehicle control (CMC) and the MCL-1 inhibitor 30 minutes before hemorrhage. Kaplan-Meier plot shows percentage of survival rate between two groups. P < 0.05 versus control.

Supplemetary file 6: The mitochondrial image of live H9c2 cells was dynamically observed by a DeltaVision Microscopy Imaging Systems. **Video S1.** Empty vector transfected H9c2 cells were treated without VPA (VPA-). **Video S2.** Empty vector transfected H9c2 cells were treated with VPA (VPA+). **Video S3.** pCMV6-AC-MCL-1 transfected H9c2 cells were treated without VPA (VPA-). **Video S4.** pCMV6-AC-MCL-1 transfected H9c2 cells were treated with VPA (VPA+).

Video S5. Non-targeting siRNA transfected H9c2 cells were treated without VPA (VPA-). **Video S6.** Non-targeting siRNA transfected H9c2 cells were treated with VPA (VPA+). **Video S7.** MCL-1 siRNA transfected H9c2 cells were treated without VPA (VPA-). **Video S8.** MCL-1 siRNA transfected H9c2 cells were treated with VPA (VPA+). **Video S9.** Tim50 siRNA transfected H9c2 cells were treated without VPA (VPA-). **Video S10.** Tim50 siRNA transfected H9c2 cells were treated with VPA (VPA+).

Supplemetary file 7: Supplemental Methods.

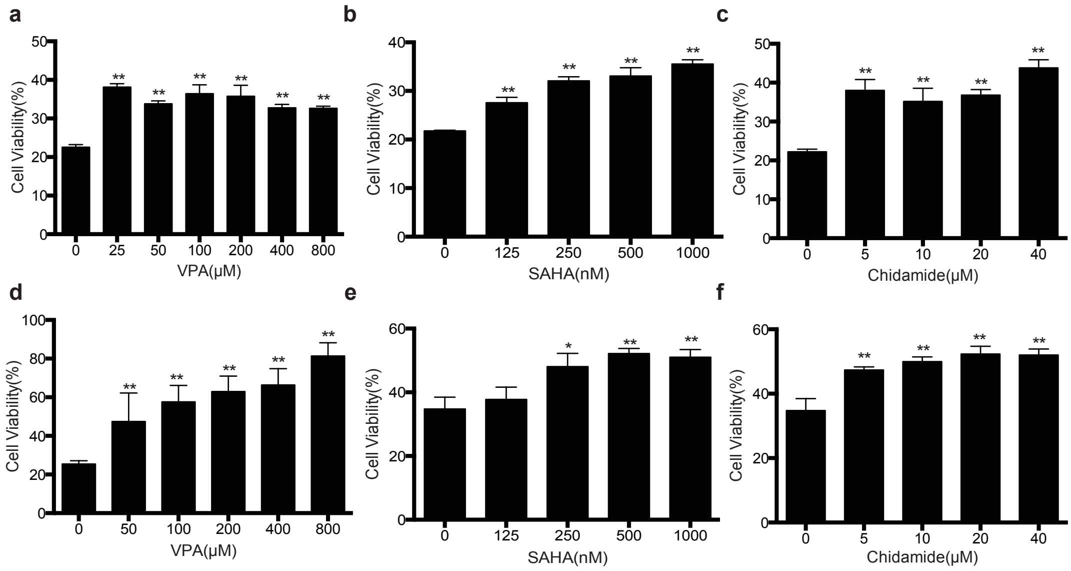

Figure S1. Effect of HDACIs treatment in cell hypoxia and oxidative stress models. (**a-c**) The myocardial protection of VPA, SAHA, chidamide was explored in the $CoCl_2$-induced hypoxia model. (**d-f**) The myocardial protection of VPA, SAHA, chidamide was explored in the H_2O_2-induced free radical model (n=3). Values are reported as means ± SD. **P* < 0.05, ***P* < 0.01 versus 0 control.

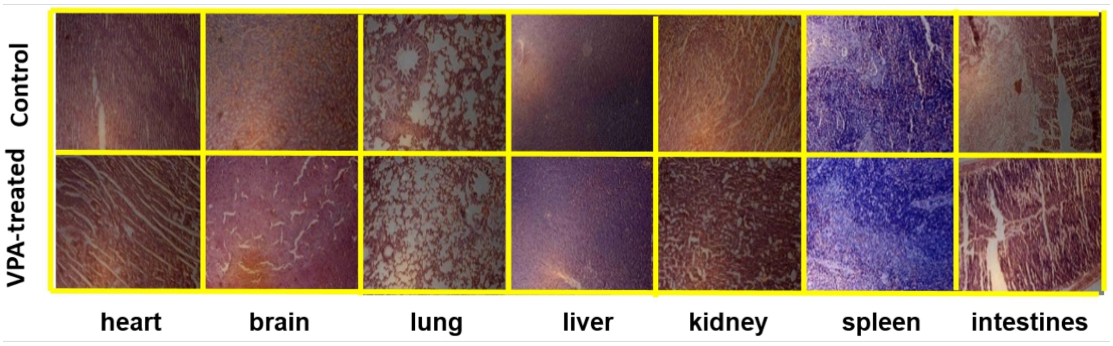

Figure S2. Histology of tissues at the endpoint of observation time in hemorrhagic rats. Representative histology views (H&E Stain) in VEH control and VPA-treated animals are shown, 100×.

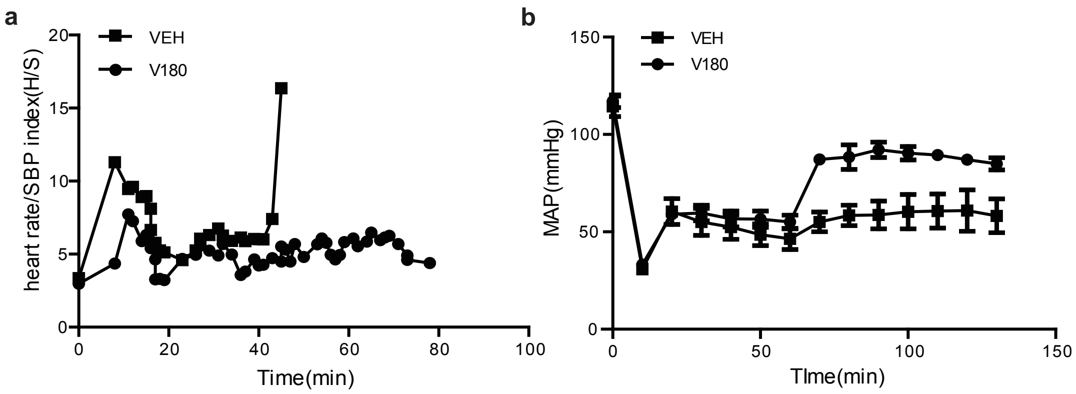

Figure S3. H/S index and MAP in rats. (**a**) 60% of total blood was removed and the rat was treated at 60 minutes; the V180 rat (n=1) was treated with 180 mg/kg VPA at 10 minutes; the VEH rat (n=1) was treated with an equal volume of NS (normal saline, 0.9% NaCl); (**b**) 60% of total blood was removed in 60 minutes; the VPA group (n=4) was treated with 180 mg/kg VPA after 10 minutes from baseline (T=0); the VEH group (n=4) was treated with an equal volume of NS.

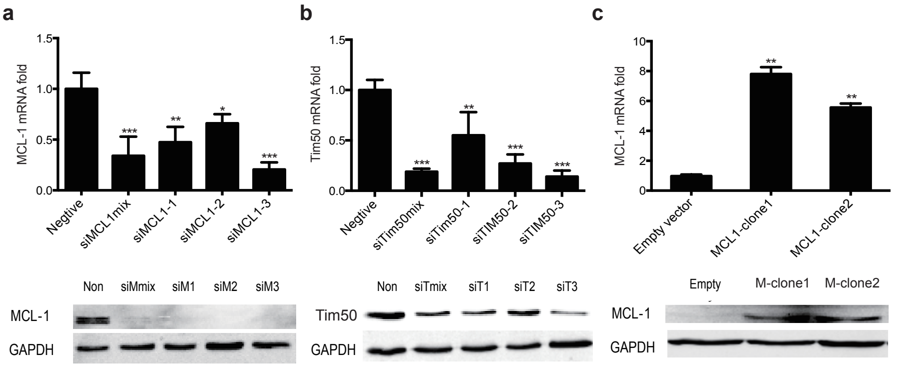

Figure S4. Knockdown and overexpression. (**a**, **b**) Gene and protein expression after RNA-mediated interference (RNAi)-gene silencing of MCL-1 and Tim50. Top: Relative gene expression was measured by qRT-PCR (n=3). Values are reported as means ± SD. **P* < 0.05, ***P* < 0.01, ****P* < 0.001 versus negative. Bottom: Protein expression was detected by Western blot. (**c**) pCMV6-AC-MCL-1 was overexpressed in H9c2 cells. Top: Relative gene expression was measured by qRT-PCR (n=3). Values are reported as means ± SD. ***P* < 0.01 versus Entry. Bottom: Protein expression was detected by western blot.

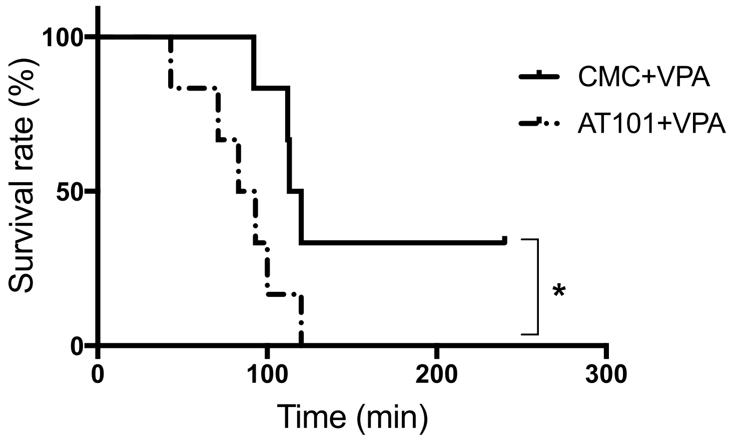

Figure S5. The ability of MCL-1 to mediate the protective activity of VPA. For the type I model, rats were treated with vehicle control (CMC) and the MCL-1 inhibitor 30 minutes before hemorrhage. Kaplan-Meier plot shows percentage of survival rate between two groups. $P < 0.05$ versus control.

The mitochondrial image of live H9c2 cells was dynamically observed by a DeltaVision Microscopy Imaging Systems. **Video S1.** Empty vector transfected H9c2 cells were treated without VPA (VPA-). **Video S2.** Empty vector transfected H9c2 cells were treated with VPA (VPA+). **Video S3.** pCMV6-AC-MCL-1 transfected H9c2 cells were treated without VPA (VPA-). **Video S4.** pCMV6-AC-MCL-1 transfected H9c2 cells were treated with VPA (VPA+). **Video S5.** Non-targeting siRNA transfected H9c2 cells were treated without VPA (VPA-). **Video S6.** Non-targeting siRNA transfected H9c2 cells were treated with VPA (VPA+). **Video S7.** MCL-1 siRNA transfected H9c2 cells were treated without VPA (VPA-). **Video S8.** MCL-1 siRNA transfected H9c2 cells were treated with VPA (VPA+). **Video S9.** Tim50 siRNA transfected H9c2 cells were treated without VPA (VPA-). **Video S10.** Tim50 siRNA transfected H9c2 cells were treated with VPA (VPA+).

Supplemental Methods

Cell damage and HDACIs rescue

In the hypoxia model, rat cardiomyoblasts H9c2 were cultured with 2 mM $CoCl_2$ (Sigma-Aldrich, St. Louis, MO, USA) for oxygen deprival for 5 hours and then treated with one of the following HDACI clinical drugs, VPA (Sigma-Aldrich), SAHA (Selleckchem, Houston, TX, USA), chidamide (Chipscreen, Shenzhen, Guangdong, China) for 5 hours. To induce oxidative stress injury, cells were treated with 0.03% H_2O_2 for 15 minutes, and then cultured with HDACI drugs for 24 hours.

Animal studies

Animals

The protocol was approved by the Animal Ethics Committee of Beijing Institute of Transfusion Medicine. Male Wistar rats (7-8 weeks old, 255-265 g) were obtained from the Vital River Laboratory Animal Technology Company (Beijing, China) and given free access to a pellet diet and purified water ad libitum. Experiments were performed after an acclimatization period of 4 days.

Surgery

The rats were anesthetized with pentobarbital sodium, then two polyethylene catheters (0.1-mm diameter) filled with heparinized saline were annulated into the left femoral artery and vein to allow for continuous recording of arterial blood pressure, collection of blood samples, and controlled bleeding. MAP and ECG readings were continuously monitored with a polygraph (Biopac Systems, Inc., M150A). The rats were observed for 10 minutes after surgery to ensure stability of the MAP and ECG. Body temperature was carefully maintained at 37 ± 0.5ºC using a heating pad (Softron, TMS-201). MAP was monitored at a minimum of 30 minutes after hemorrhage until

the rats showed initial signs of recovery from anesthesia, and then the femoral catheters were removed. The skin over the incision sites was carefully closed by sutures and covered with antibiotic ointment. OCR was measured using the Seahorse XFe96 analyser. After the hemorrhage procedures were completed, the rats were kept under observation in heated cages at 37°C with free access to diet pellets and purified water. Exclusion criteria were as follows: MAP < 100 mmHg before hemorrhage, surgical bleeding > 0.5 ml during catheterization, and surgical time > 15 minutes.

Hemorrhage procedures

Hemorrhage was performed using adjustable pumps (Lange Corporation). For phase 1 (P1) hemorrhage, 40% volume of whole blood was rapidly withdrawn from the femoral artery over 10 minutes. For phase 2 (P2) hemorrhage, the femoral vein was allowed to bleed another 20% of total blood over the next 50 minutes to simulate the process of a chronic bleeding stage. Animals were randomly assigned to different groups; VPA was dissolved in 0.9% saline (250 μl) and administered through the right femoral vein. The first hemorrhage model included groups treated at the end of P1, the second hemorrhage model treated at the end of P2.

Groups

The first hemorrhage model included groups treated with VPA at the end of P1 (10 min treatment groups), classified as follows: Sham (catheterization without hemorrhage, n=12), control group (no treatment after hemorrhage, n = 12), VEH group (0.9% saline 250 μL, n = 12), V60 group (VPA 60 mg/kg, n = 12), V120 group (VPA 120 mg/kg, n = 12), V180 group (VPA 180 mg/kg, n = 12), pretreat group (VPA 180 mg/kg, n = 12, i.m. 24 hours before surgery), CMC+V180 group (CMC, VPA 180 mg/kg, n = 6) and AT101+V180 group (AT101 60mg/kg, VPA 180 mg/kg, n = 6). Rats were treated with vehicle control (CMC, Sigma-Aldrich) and AT-101

(Selleckchem) dissolved in CMC (60 mg/kg) by oral gavage 30 minutes before the beginning of hemorrhage. VPA or saline was injected through the femoral vein other than the pretreat group, in which VPA was administered intramuscularly 24 hours before surgery. The second hemorrhage model included groups treated at the end of P2 (1 h treatment groups), classified as follows: Sham (catheterization without hemorrhage, n=12), control group (no treatment after hemorrhage, n = 12), VEH group (0.9% saline 250 μL, n = 13), V60 group (VPA 60 mg/kg, n = 12) and V180 group (VPA 180 mg/kg, n = 11).

Loss-of-function and gain-of -function experiments

siRNA knockdown

MCL-1 and Tim50 knockdown cells were established for loss-of-function experiment. H9c2 cells in Opti-MEM® I Reduced Serum Medium (Invitrogen, Carlsbad, CA, USA) were transfected with 60nM siRNA (Sigma-Aldrich) specific for rat MCL-1 or Tim50 using Lipofectamine® RNAiMAX Reagent (Invitrogen). The efficiency of gene knockdown in H9c2 cells were verified by real-time RT-PCR and Western Blot after 48-hour incubation. siRNA used were as follows: MCL-1, 5’-CCAAGAAAGCUGCAUCGAAdTdT-3’, 5’-GAUUAUAACACGGCAGAAAdTdT-3’ and 5’-GUCUGAAUGAAGCAAAGUUdTdT-3’; and TIM50, 5’-GGACUGUCAGCAUCGUCUAdTdT-3’, 5’-CGCACUCCAGAACAGGUUAdTdT-3’ and 5’-GACCCAAGGGCCACAGCAUdTdT-3’.

MCL-1 over-expression

MCL-1 over-expression cells were established for gain-of-function experiment. Briefly, H9c2 cells were transfected with full-length wild-type MCL-1 expression

plasmid pCMV6-AC-MCL-1 (OriGene, Rockville, MD, USA) by LipofectamineTM2000 (Invitrogen), and subsequently selected for G418 resistance after 48 hours of plasmid infection. PCMV6-Entry transfected cells were used as negative controls. The over-expression of MCL-1 was confirmed by real-time qRT-PCR and Western blotting analysis for mRNA and protein expression, respectively.

Real-time RT-PCR

RNA was extracted with TRIzol® Reagent (Invitrogen). Total RNA (1 μg) was reverse transcribed using random primers and GoScript™ Reverse Transcription System (Promega). Real-time PCR was performed on an IQ5 Real-Time PCR Detection System (BioRad) with GoTaq® qPCR Master Mix (Promega) for MCL-1, Tim50 and β-actin transcripts. The primers: MCL-1, forward 5'-TTTCTTTTGGTGCCTTTGTG-3', reverse 5'-ATCCCAGCCTCTTTGTTTCAC-3'; Tim50, forward 5'-CAACAGTGAGCGAGAGAAACAG-3', reverse 5'-GCTGGGGATGTCAAAACTAAGA-3'; and β-actin, forward 5'-CACCCGCGAGTACAACCTTC-3', reverse 5'-CCCATACCCACCATCACACC-3'. Product sizes are 137 bp, 236 bp and 207 bp, respectively.

Western blotting and immunoprecipitation

For immunoblot analysis, rat tissues and cells were lysed in a lysis buffer (50 mM Tris at pH7.4, 150 mM NaCl, 1% Triton X-100, 1 mM EDTA) and protease inhibitor cocktail (Roche) on ice. Lysates were immunoblotted or immunoprecipitated with anti-MCL-1, anti-LDH-A rat monoclonal antibodies or rat IgG (Santa Cruz Biotechnology) and protein A/G agarose (Santa Cruz Biotechnology). The

antigen/antibody complexes were visualized using the Odyssey® Infrared Imaging System (LI-COR Biosciences). Plasmids were pCMV6-MCL-1/Entry (OriGene Biotechnology). The following antibodies were used for Western Blot assay: anti-MCL-1 (Abcam, Cambridge, MA, USA), anti-Tim50 (Abcam) and GAPDH (Cell Signaling, Danvers, MA, USA).

Oxygen consumption rate assay

A Seahorse Bioscience Instrument (Seahorse Bioscience, North Billerica, MA, USA) was used to measure oxygen consumption rate as per manufacturer's instructions. $1x10^4$ cells (MCL-1 or Tim50 siRNA and control cells) per well were plated on 96 well XF plate and grown in standard DMEM medium with 15% FBS, incubating overnight at 37°C in 5% CO_2 atmosphere. For oxygen consumption rate measurement, growth medium was replaced with XF assay medium. The cells were pre-incubated at 37°C for 1 hour before starting the analysis. Oligomycin was used at a final concentration of 2μM, FCCP at 0.5μM, Antimycin A and Rotenone at 0.5μM.

Mitochondrial microscopy imaging

MCL-1 over-expression and siRNA knockdown H9c2 cells were cultured in DMEM supplemented with 15% FBS and incubated overnight. The cell culture media was then replaced by hypoxic medium (obtained by DMEM media supplement with 2 mM CoCl2 solution) and cultured for additional 6h to induce hypoxic injury. PCMV6-Entry and non-targeting siRNA transfected H9c2 cells were applied as control respectively. The hypoxic medium was replaced by the serum-free pre-warmed media containing Mitochondrion-Selective Probes (MitoTracker® Green

FM), incubation for 30 minutes under cell growth conditions. After staining is complete, replace the staining solution with fresh prewarmed media with or without VPA, and observe cells using a DeltaVision Microscopy Imaging Systems (Applied Precision) using 10 min frames during 12 hours.
